# The association of *γδ*T lymphocytes with cystic leukomalacia in premature infants

**DOI:** 10.3389/fneur.2022.1043142

**Published:** 2022-12-02

**Authors:** Mengjie Yuan, Xinyun Jin, Fanyue Qin, Xiaoli Zhang, Xiaoyang Wang, Enwu Yuan, Ying Shi, Falin Xu

**Affiliations:** ^1^Department of Neonatology, The Third Affiliated Hospital of Zhengzhou University, Zhengzhou, China; ^2^Department of Academy of Medical Sciences of Zhengzhou University, Zhengzhou, China; ^3^Henan Key Laboratory of Child Brain Injury and Henan Pediatric Clinical Research Center, Third Affiliated Hospital and Institute of Neuroscience of Zhengzhou University, Zhengzhou, China; ^4^Advanced Medical Research Center of Zhengzhou University, Zhengzhou, Henan, China; ^5^Department of Clinical Laboratory, The Third Affiliated Hospital of Zhengzhou University, Zhengzhou, China

**Keywords:** preterm, cPVL, T lymphocytes, *γδ*T, outcome

## Abstract

**Background:**

Periventricular leukomalacia (PVL) is an essential cause of cerebral palsy in preterm infants, and cystic PVL (cPVL) is the most severe form of the disease. The pathogenesis of cPVL is complex, and immune imbalances and inflammatory responses may play an essential role in it.

**Objective:**

*This study aimed* to investigate the correlation between peripheral blood lymphocyte subsets, especially *γδ*T cells with the pathogenesis of cPVL in preterm infants.

**Methods:**

Peripheral blood from preterm infants with GA < 32 weeks and BW < 1,500 g was used in this study and was collected at 34 weeks corrected gestational age and within 24 h after the diagnosis with cranial MRI or cranial ultrasound. The infants were divided into cPVL groups and control groups. Flow cytometry was used to detect peripheral blood *γδ*T, CD3+, CD4+, CD8+, and the proportion of total lymphocytes. Multiplex cell assays were used to detect the concentration of extracellular serum cytokines IL-6, IL-2, IL-8, IL-17A, IL-10, IL-1RA, eotaxin (CCL11), MCP-1 (CCL2), CXCL1, G-CSF, and IFN*γ*. A follow-up visit was carried out when the patient was 3 years old.

**Results:**

After correcting for confounding factors, the proportion of peripheral blood *γδ*T in the cPVL group was significantly lower than that in the control group (β: 0.216; 95% CI: 0.058–0.800, *P* < 0.022). Peripheral blood *γδ*T (AUC: 0.722, *P*=0.006) and multivariate binary regression model (AUC: 0.865, *P* < 0.000) have good diagnostic values for cPVL. Peripheral blood *γδ*T has some predictive power for neurodevelopmental outcomes in preterm infants (AUC: 0.743, *P* = 0.002).

**Conclusion:**

It seems that peripheral blood *γδ*T cells are inversely correlated with cPVL, which is not only a risk factor for cPVL disease but also neurodevelopmental outcomes in preterm infants. However, the causality of cPVL and various lymphocytes is unclear and needs further study.

## Introduction

White matter injury (WMI) is the main type of brain injury in preterm infants, including periventricular leukomalacia (PVL) and disseminated white matter injury, of which cystic PVL (cPVL) is the most serious. Survivors often have different degrees of neurological sequelae, including cerebral palsy, visual impairment, cognitive, behavioral, and attention deficits (1–3). The pathogenesis of brain injury in preterm infants is complex, and perinatal asphyxia and inflammation are the main risk factors for neurological injury in preterm infants (4). Neurological disorders in the neonatal period may be associated with elevated systemic inflammation, and although there are many factors contributing to poor prognosis in preterm infants, chronic inflammation has been identified as a potentially important cause of brain injury and neurodevelopmental disorders (5). Studies showed many complications of preterm infants were associated with immune dysfunction and imbalance (6, 7). Currently, the diagnosis of cPVL mainly relies on cranial ultrasound and cranial MRI, with fewer studies on serum biomarkers.

The role of T lymphocyte and its relevant cytokine-mediated inflammation in brain injury in preterm infants has gradually attracted people's interest. Studies have reported that T lymphocytes in preterm infants with brain injury have changed, and the secretion of various cytokines has changed (8–10). *γδ*T cells are a type of T lymphocyte that carries *γδ*TCR, which also differs from αβT cells in terms of ontogenesis and anatomical localization. These cells are usually derived from the fetal or neonatal thymus gland and migrate into mucosal tissue at an embryonic stage. As a result, they respond rapidly to infection and tissue damage (11). *γδ*T cells can also utilize NK cell receptors and respond as native immune cells, which is an essential subset of “unconventional” T lymphocytes, linking innate and adaptive immunity (12–14). Currently, immune function in preterm infants is unclear, which may be associated with deficiencies in T lymphocyte activation and cytokine production (15). In animal models, Zhang et al. (16) have found that *γδ*T cells rather than αβT cells contribute to sepsis-induced white matter damage and subsequent early motor dysfunction. However, in recent years, there have been fewer studies on the correlation between peripheral blood *γδ*T cells and cPVL (17). In this study, we explored the *γδ*T lymphocyte subset and related cytokine levels in the peripheral blood of preterm infants with cystic cerebral softening, aimed at finding a sensitive, simple, serum biological marker that reflects brain injury.

## Materials and methods

### Ethics statement

This study was approved by the Ethics Committee of the third affiliated hospital of Zhengzhou University (Ethics number: 2021-027-01). Informed consent for publication has been obtained from the parents or guardians of the patients.

### Patients

#### Inclusion and exclusion criteria

**Figure 1:**
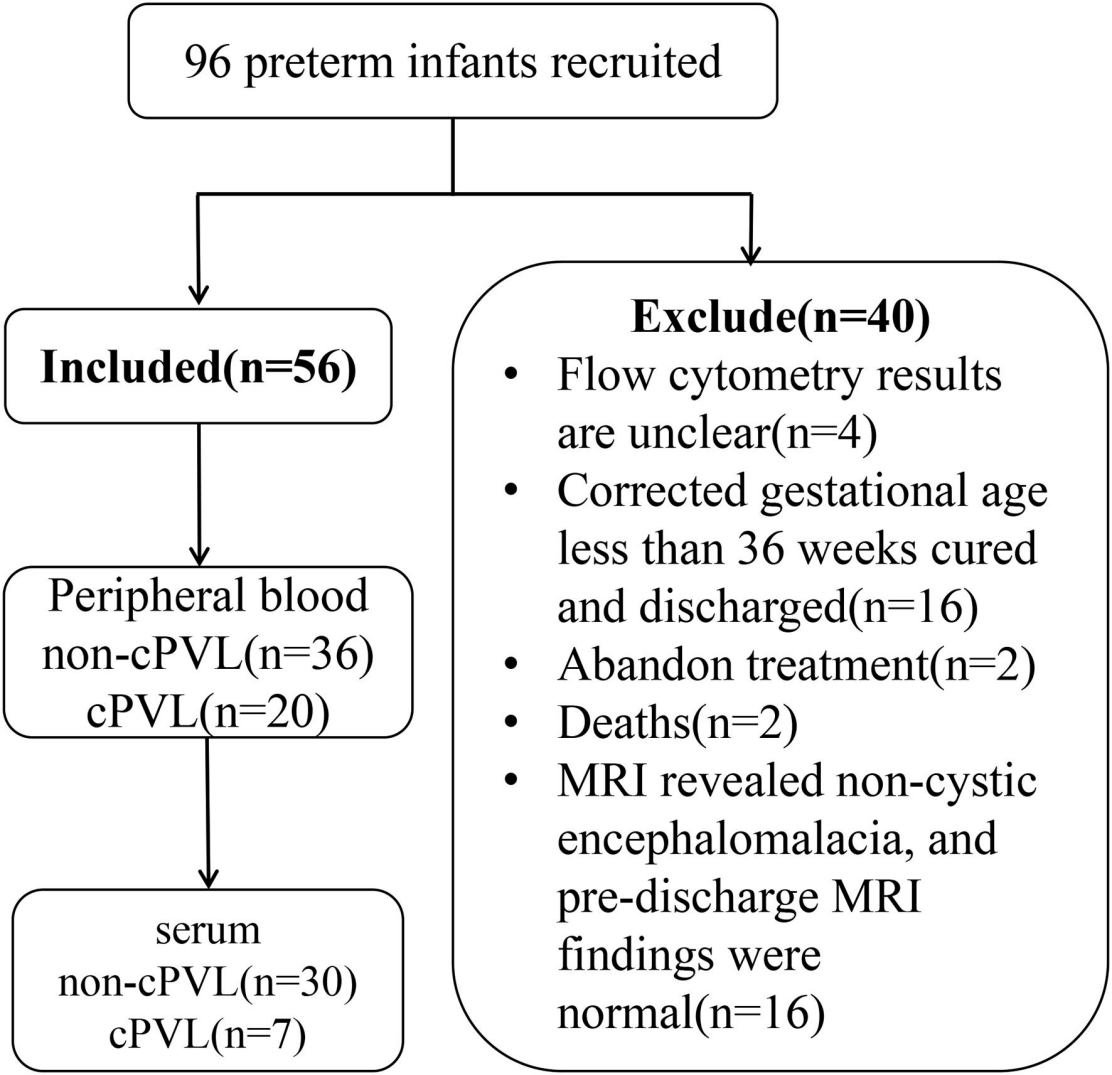
Flow chart of the study participants. cPVL, cystic peripheral ventricular leukomalacia.

Ninety-six premature infants were recruited from the neonatal intensive care unit (NICU) of the Third Affiliated Hospital of Zhengzhou University from April 2019 to May 2021, as shown in Figure 1. We performed cranial ultrasound scans of all newborns within 72 h of birth and repeated scans at a 7-day interval until discharge. If the cranial ultrasound showed abnormalities, a cranial MRI is performed to confirm the diagnosis of brain injury.

Peripheral blood from preterm infants with GA < 32 weeks and BW < 1,500 g was used for the study, collected at 34 weeks corrected gestational age and within 24 h after the diagnosis with cranial MRI or cranial ultrasound. The diagnosis of PVL was made based on the basis of MRI findings, routinely performed on the basis of Volpe's study (18). According to the pathological type and radiographic manifestations of PVL, the first confirmed PVL in grade II-IV after birth is cystic periventricular leukomalacia (cPVL) (19).

Congenital malformations, intraventricular hemorrhage (IVH) in grades 3 and 4, inherited metabolic diseases, polycythemia, thrombocytopenia, blood transfusions 1 week before sampling, infection, mothers with immune disorders, newborns who abandoned treatment or died within 14 days of birth, and preterm infants who lack imaging data were excluded.

We followed these participants until the age of 3 years when the neurodevelopment of the children was assessed by a physician specialized in outpatient care using the Gesell Developmental Diagnosis Scale (GDDS). There are five domains in the Chinese version of the GDDS including domains of adaptability, gross motor, fine motor, language, and social-emotional responses (20). The development quotient (DQ) of each domain was calculated for each participant. According to the full-scale DQ, the development of infants was classified as follows: normal (DQ ≥ 85), deficient (DQ < 75), and borderline (75 ≤ ~ < 85). DQ in any single domain falling below 75 was also considered as growth retardation. The child was assessed for cerebral palsy according to the international diagnostic criteria for cerebral palsy (21).

### Data collection

#### Blood collection

Premature infants with cPVL were selected as the study group, and premature infants with similar gestational age (±1 week) and birth weight (±100 g) at the same time were selected as the control group. About 1 ml of peripheral venous blood was collected and stored in a vacuum heparin sodium anticoagulant tube at room temperature. All flow cytometry tests were performed within 8 h of blood collection. Considering that is unethical to collect large quantities of blood from preterm infants for research purposes only, we tracked the children's laboratory results on the day we diagnosed the cPVL and recovered 50 ul of serum from the Laboratory Department. The samples were stored at −80°C in a freezer and used for the cytokine assay by the Luminex method. Unfortunately, we only recovered serum from seven cases of cPVL and 30 controls.

#### Flow cytometry analysis

About 100 uL of whole anticoagulant blood was taken into the sample tube.

Cell surface markers: The following anti-human monoclonal antibody kits, including CD3 APC-H7 (clone SK7), CD4 BV605 (clone RPA-T4), CD8 PE-Cy7 (clone M5E2), TCR*γδ* BV421 (clone B1), and TCRαβ FITC (clone T10B9.1A-31), were used, and then, the whole blood was incubated avoiding light for 20 min at room temperature. After the incubation, 2 ml of 1X erythrocyte lysis buffer (1 (10X erythrocyte lysis buffer): 9 (dH2O); BD Pharmingen) was added at room temperature for 8–12 min, until the cell suspension was translucent, and centrifuged at 500 g for 5 min; then, the cells were washed, and the supernatant was discarded. Then, 1 ml of phosphate-buffered saline (PBS) was added and centrifuged at 500 g for 5 min again. (All anti-human monoclonal antibodies purchased from eBioscience, using the concentration recommended by the manufacturer).

**Figure 2:**
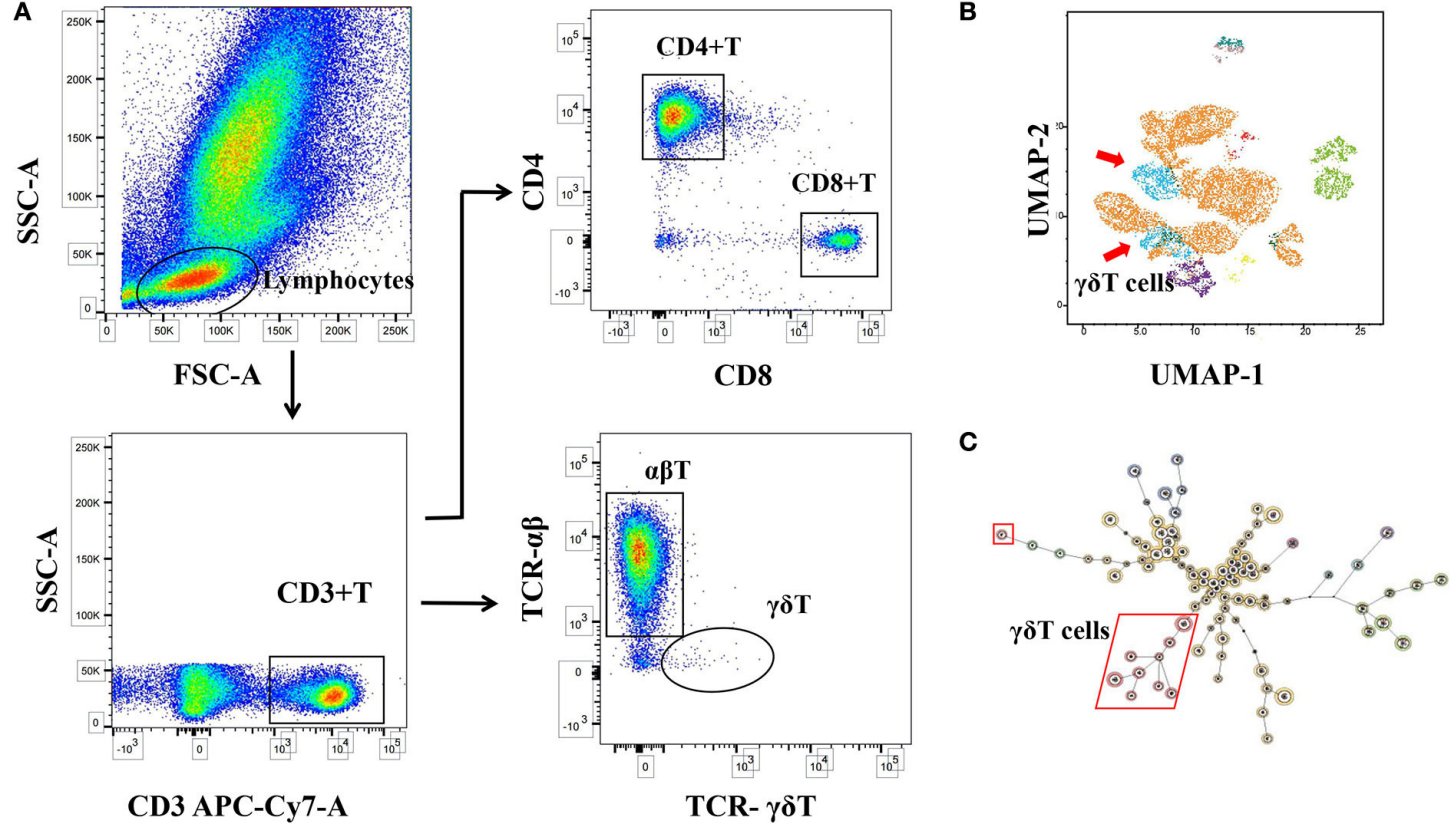
Logic diagram for immune cell analysis. **(A)** Circle gate logic of T-cell subsets detected by flow cytometry. **(B)** Maps of self-organizing and consistent hierarchical clustering of different T-cell subsets (FlowSOM). **(C)** Dimensionality reduction profile of immune cells (Uniform Manifold Approximation and Projection, UMAP). *γδ*T cell differences were found to be significant.

Flow cytometry was performed using the FACSCanto II flow cytometer (BD Biosciences, BD-FACSCanto II). The data were analyzed using FACS-Diva software (TreeStar, BD FACSDiva 8.0.1, BD Biosciences) and FlowJo software (Ver10.8.1) (refer to Figure 2). To ensure the accuracy of the data, the quality control of the instrument, the processing and detection of specimens, and the analysis of the data were all operated by the same experimenter.

#### Luminex multifactor cell assay

Human ProcartaPlex Mix&Match 11-plex (Invitrogen by Thermo Fisher Scientific) was used to detect the secretion of cytokines related to *γδ*T in serum. The instrument used for the detection was the Luminex^®^200TM system and performed according to the requirements of the reagent manufacturer. Serum samples were thawed on ice and mixed well by vortexing followed by centrifugation at 10,000 × g for 10 mins. Cytokine concentration (pg/ml) in the serum samples was determined by comparing it to the standard curve prepared on each plate. The ProcartaPlex Analysis was used to analyze the transformation of cytokine concentrations. All tests were set up with three parallel controls, and finally, the average was taken for calculation.

### Statistical analysis

Statistical analysis was performed using SPSS 26.0 and R version 3.6.3 software. The characteristics of all subjects were expressed in terms of the number of categorical variables (percentages), *the mean* ± *standard* deviation of the variables with parametric distribution, or the median (*inter-quartile range)* of the non-parametric distribution variables. Data were tested for normality using a Shapiro–Wilk normality test before running statistical tests, Student's *t-*test was used for data showing normal distribution, and the Mann–Whitney *U*-test was used for data that did not meet the normal distribution. For categorical variables, the chi-square tests were used for comparisons.

Univariate and multivariate logistic regression models were used to compare *γδ*T lymphocyte subsets and related cytokine levels in the cPVL and control groups. Confounding factors adjusted in multivariate linear regression models include the effects of asphyxia (Model 1) and premature rupture of membrane (Model 2) on *γδ*T. In the univariable analysis, the risk variables of *P*<*0.05* and risk factors for cPVL that were reported in the literature including asphyxia, RDS, prenatal steroid use, premature rupture of membranes, and placental abnormality were selected (7, 22), and the stepwise multiple logistic regression analysis was used to screen out the risk factors of cPVL, constructing a nomogram, and used the receiver operating characteristic curve (ROC) to analyze the sensitivity of the model. The calibration curve evaluates the calibrated degree of the model, and the clinical practicability of the model was checked by decision curve analysis (DCA). Finally, the predictive value of *γδ*T cells for neurodevelopmental outcomes was used to train the ROC curve. *A two-tailed p-value of 0.05* was considered significant at the *95% confidence interval (CI)* level.

## Results

### Comparison of the general situation, perinatal factors, and complications of the two groups of premature infants

The general situation of the study population and perinatal-related factors and complications are shown in Table 1. There were no significant differences between the cPVL and control groups in terms of birth weight, gestational age, gender, and other factors (*P* > 0.05), and there were statistically significant differences in postnatal 1 min Apgar scores, 5 min Apgar scores, asphyxia, intraventricular hemorrhage, ROP, and placental abnormalities (*P* < 0.05).

**Table 1:** Comparison of general conditions and risk factors in the study population.

**Group**	**Control**	**Cystic PVL**	**P-value**
	**(*n* = 36)**	**(*n* = 20)**	
Sex (male)	22 (0.61)	15 (0.75)	0.293
Gestational age	29.94 ± 1.98	29.03 ± 2.14	0.714
Birth weight	1.28 (1.08–1.43)	1.325 (0.937–1.63)	0.851
APS 1 min	1–3 1 (0.03)	1–3 1 (0.05)	0.004**
	4–7 7 (0.19)	4–7 12 (0.6)	
	8–10 28 (0.78)	8–10 7 (0.35)	
APS 5 min	4–7 3 (0.08)	4–7 6 (0.3)	0.039^*^
	8–10 33 (0.92)	8–10 14 (0.7)	
C/S	27 (0.75)	14 (0.70)	0.686
Antenatal steroids	31 (0.86)	14 (0.70)	0.146
HDP	10 (0.28)	3 (0.15)	0.278
GDM	5 (0.14)	4 (0.20)	0.551
Maternal age, years	31.55 ± 4.05	30.78 ± 5.02	0.438
Amniotic fluid anomaly	5 (0.14)	5 (0.25)	0.298
PROM	10 (0.28)	9 (0.45)	0.192
Placental abruption	2 (0.06)	5 (0.25)	0.035^*^
Didymous	5	7	0.065
Triplets	2	2	0.536
IUGR	2 (0.06)	1 (0.05)	0.903
Asphyxia of newborn	11 (0.31)	12 (0.60)	0.032^*^
NRDS	34 (0.94)	17 (0.85)	0.235
PDA	20 (0.56)	12 (0.60)	0.747
Apply ibuprofen	1 (0.03)	1 (0.05)	0.668
Cephalomeningitis	1 (0.03)	2 (0.10)	0.25
Septicemia	9 (0.25)	7 (0.35)	0.427
PVH-IVH	4 (0.11)	11 (0.55)	0.000***
Hypothyroidism	1 (0.02)	3 (0.15)	0.089
Pneumonia	9 (0.25)	10 (0.50)	0.058
Neonatal cholestasis	6 (0.17)	8 (0.40)	0.053
PPHN	0 (0)	1 (0.05)	0.176
Surgical NEC	2 (0.06)	3 (0.15)	0.235
BPD	15 (0.42)	6 (0.30)	0.388
ROP	1 (0.03)	5 (0.25)	0.010^*^
Pneumorrhagia	3 (0.08)	5 (0.25)	0.088

APS, Apgar score; C/S, cesarean section; HDP, hypertensive disorders of pregnancy; GDM, gestational diabetes mellitus; PROM, premature rupture of membrane; IUGR, intrauterine growth restriction; NRDS, neonatal respiratory distress syndrome; PDA, patent ductus arteriosus; PVH-IVH, periventricular-intraventricular hemorrhage; PPHN, persistent pulmonary hypertension of newborn; NEC, necrotizing enterocolitis; BPD, bronchopulmonary dysplasia; ROP, retinopathy of prematurity.

* Indicates *P* < 0.05. The diagnostic criteria for neonatal asphyxia are Apgar less than 7 points. The ^***^ symbol indicates the values of *P* < 0.001 and the ^**^ symbol indicates the values of *P* < 0.01.

### Comparison of the distribution of peripheral blood T lymphocyte subsets and related cytokine concentrations of cPVL and control groups

**Figure 3:**
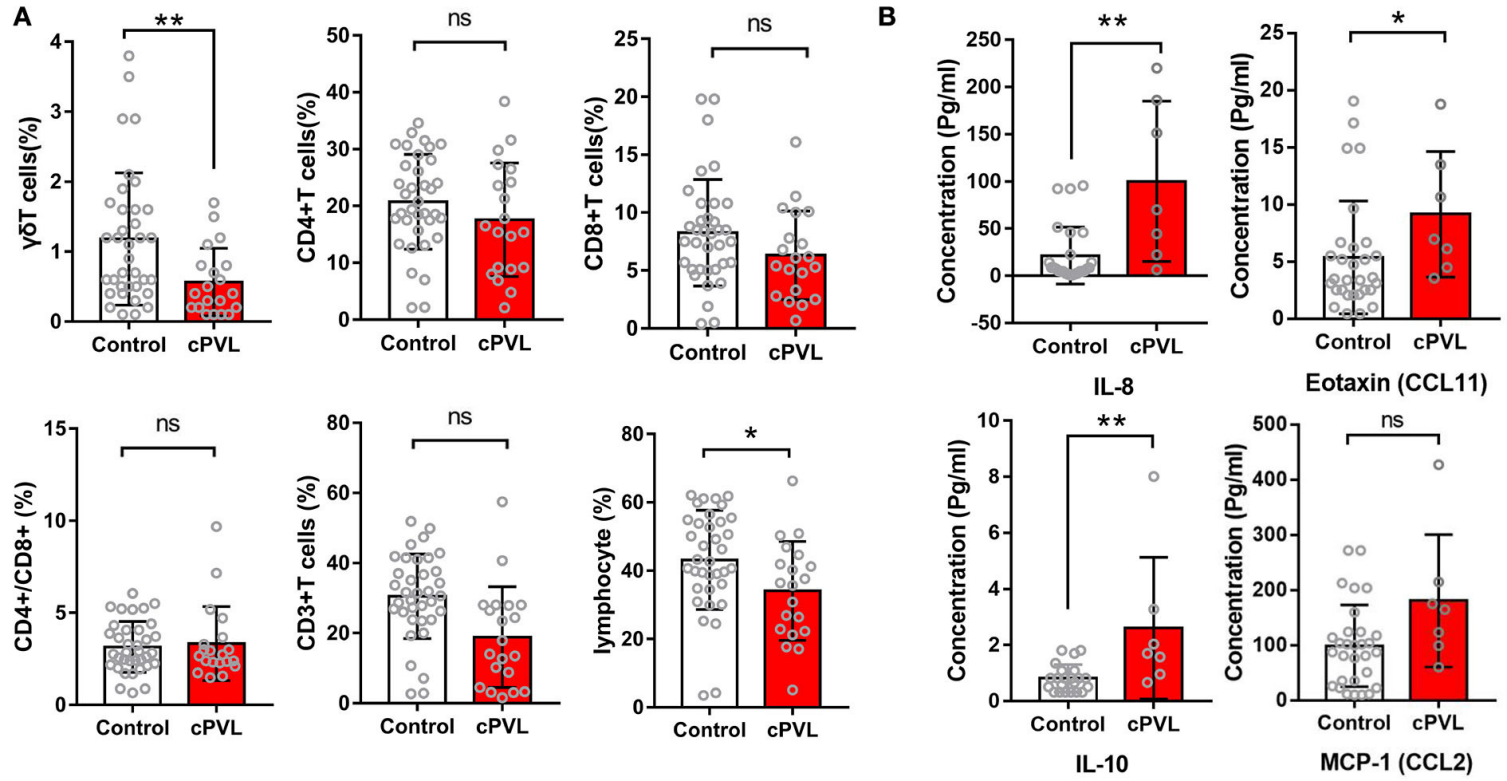
Distribution of peripheral blood T-cell subsets is within the lymphocyte population and serum cytokine concentrations in two groups of preterm infants. **(A)** Dot bar graphs showing the percentage of *γδ*T, CD3+T, CD4+T, CD8+T, CD4+T/CD8+T, and lymphocytes cells in cPVL (*n* = 20) and control (n = 36) groups. **(B)** Dot bar graphs showing the percentage of serum concentration of IL-8, IL-10, eotaxin (CCL11), and MCP-1 (CCL2) in cPVL (*n* = 7) and control (*n* = 30) groups. A Mann–Whitney *U-*test was performed for all analyses, and a *P* < 0.05 was considered significant. ***P ≤ 0.001; **0.001 <*P* ≤0.01; *0.01 <*P* ≤0.05; ns: *P*>0.05.

There were no significant differences in the proportion of peripheral blood CD3+, CD4+, CD8+, and CD4+/CD8+ in the cPVL group and the control group (*P* > 0.05). The ratio of peripheral blood *γδ*T cells in the cPVL group was 0.57 ± 0.48, which was significantly lower than that in the control group (1.18 ± 0.94), and the difference was statistically significant (*t* = −2.734, *P* = 0.006). During statistical analysis for dot bar graphs, four controls were found to have a high percentage of *γδ*T cells, and the examination of their health status revealed no infection, meeting the study inclusion criteria, and the experimental procedure was operated without abnormalities, confirming that the differences between the cPVL and control groups were true. The proportion of total lymphocytes in the cPVL group (34.12 ± 14.47) was significantly lower than in the control group (43.18 ± 14.53), and the difference was statistically significant (*t* = −2.291, *P* = 0.022). The concentration of peripheral blood IL-8 in cPVL preterm infants was 100.19 ± 84.83 pg/ml, which was higher than the control group (21.44 ± 30.07 pg/ml), and the difference was statistically significant (*t* = −2.688; *P* = 0.007). The concentration of IL-10 was 2.61 ± 2.53 pg/ml, which was higher than that in the control group (0.82 ± 0.48 pg/ml), and the difference was statistically significant (*t* = −2.726; *P* = 0.006). The concentration of eotaxin (CCL11) was 9.15 ± 5.49 pg/ml, which was higher than that in the control group (5.36 ± 4.94 pg/ml), and the difference was statistically significant (*t* = −2.023; *P* = 0.043). There were no significant differences in the concentration of MCP-1 (CCL2) in the cPVL group and the control group (*P* > 0.05). The concentrations of IL-2, IL-17, IL-6, IFNγ, CXCL1, G-CSF, and IL-1RA were too low to be determined (refer to Supplementary Tables 1, 2, and Figure 3).

To explore the correlation between with peripheral blood *γδ*T and cPVL, we further analyzed these correlations using the linear regression models to capture trade-off between sensitivity and specificity. As shown in Table 2, after adjusting for suffocation in model 1, the proportion of *γδ*T decreased (β: −2.749; CI: −1.132~–0.177) is significantly associated with cPVL. Falling in the proportion of *γδ*T using decrease in the proportion of *γδ*T (β: −0.400; CI: −1.196~–0.223), even after continuous adjustment of asphyxia and premature rupture of membranes in model 2, it was still significantly associated with cPVL; however, after adjusting for all confounding variables, the correlation between the decrease in the proportion of *γδ*T and cPVL was no longer significant. The ROC curve was used to evaluate the ability of *γδ*T to distinguish ability of percent of *γδ*T to distinguish between cPVL and control groups (**Figure 5E**), the optimal tangent point of *γδ*T was set at the maximum value of the number of modern finger models, the optimal cut point of *γδ*T was set at the maximum value of Youden's model, and the AUC was 0.722 (95% CI 0.584–0.859), and the cut-off value was 0.85 (sensitivity 85%, specificity 52.80%).

**Table 2:** Multivariate linear logistic regression model compares the *γδ*T ratio between cPVL and controls.

	**Crude*****β*** **(95%Cl)**		**Model 1β (95%Cl)**	**Model 2β (95%Cl)**
	**Control (*n* = 36)**	**cPVL (*n* = 20)**	**cPVL (*n* = 20)**	**cPVL (*n* = 20)**
*γδ*T cells	1.00(ref)	−0.347 (1.070,1.161) *P* = 0.009**	−2.749 (−1.132, −0.177) *P* = 0.008**	−0.400 (−1.196, −0.223) *P* = 0.005**

Data were presented as β (95% CI). cPVL, cystic peripheral ventricular leukomalacia. β (95% CI), regression coefficient (95% confidence interval). Univariate linear regression was used for the unadjusted model, and multivariate linear regression was used for the adjusted model.

a. No adjustment.

b. Adjust neonatal asphyxia.

c. Adjust neonatal asphyxia and premature rupture of fetal membranes.

***P ≤ 0.001;

**0.001 <*P* ≤ 0.01;

*0.01 <*P* ≤ 0.05.

### Potential clinical utility of *γδ*T cells in clinical applications by machine learning

**Figure 4:**
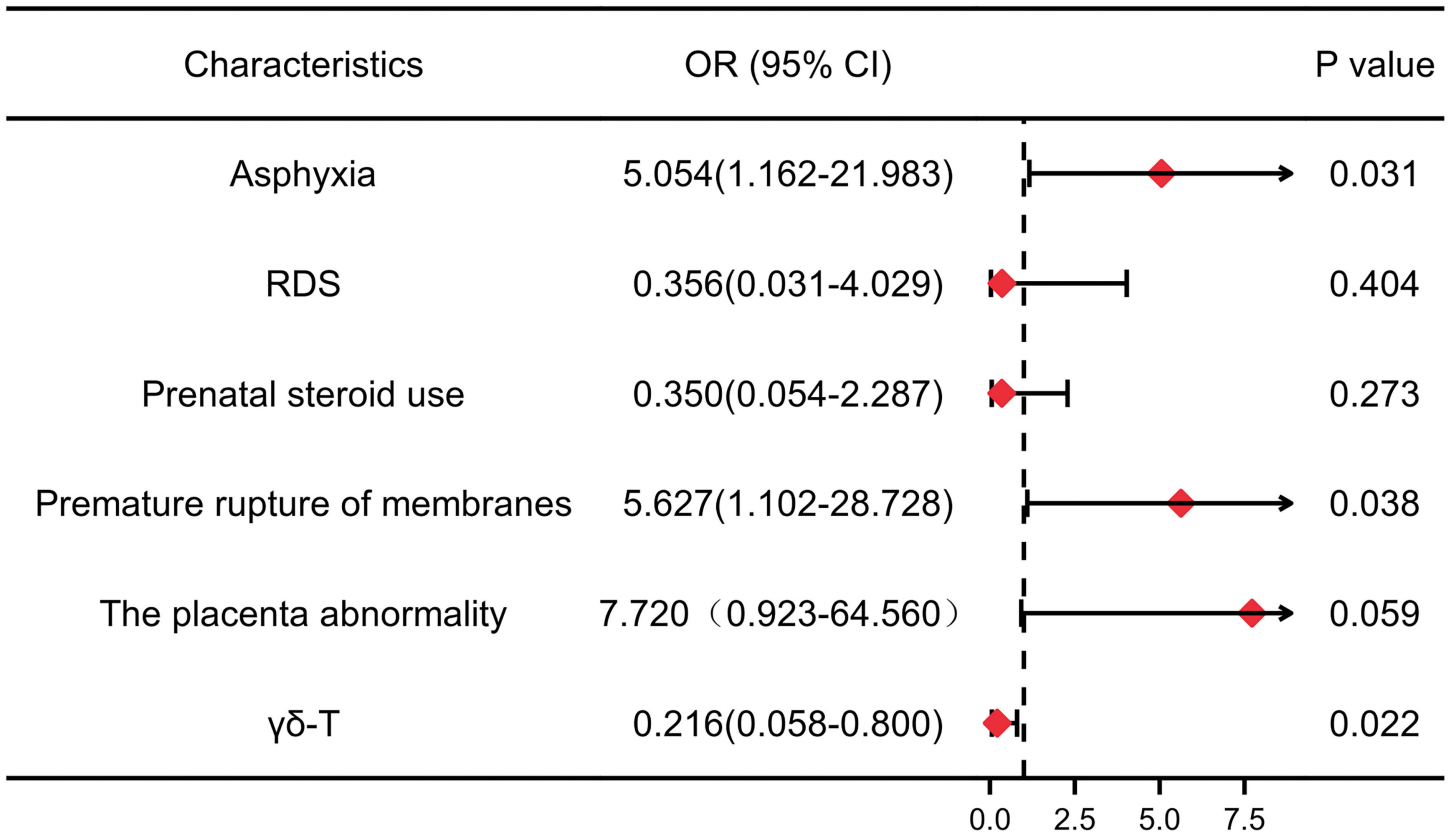
Forest plots for meta-analyses of adjusted odds ratios depicting the strength of association between risk factors and cPVL. The red rhombuses represent OR, and the horizon lines represent 95% confidence intervals. *P* < 0.05 was considered significant. OR: odds ratio. 95% CI, 95% confidence interval. RDS, respiratory distress syndrome.

Logistic regression analysis was carried out with cPVL (yes or no) as the dependent variable. A multivariate regression model was established step by step, and the confounding factors were corrected (Figure 4). The results showed that asphyxia (OR 5.054; 95% CI: 1.162–21.983) and premature rupture of membranes (OR 5.627; 95% CI: 1.102–28.728) had an increased risk of developing cPVL.

**Figure 5:**
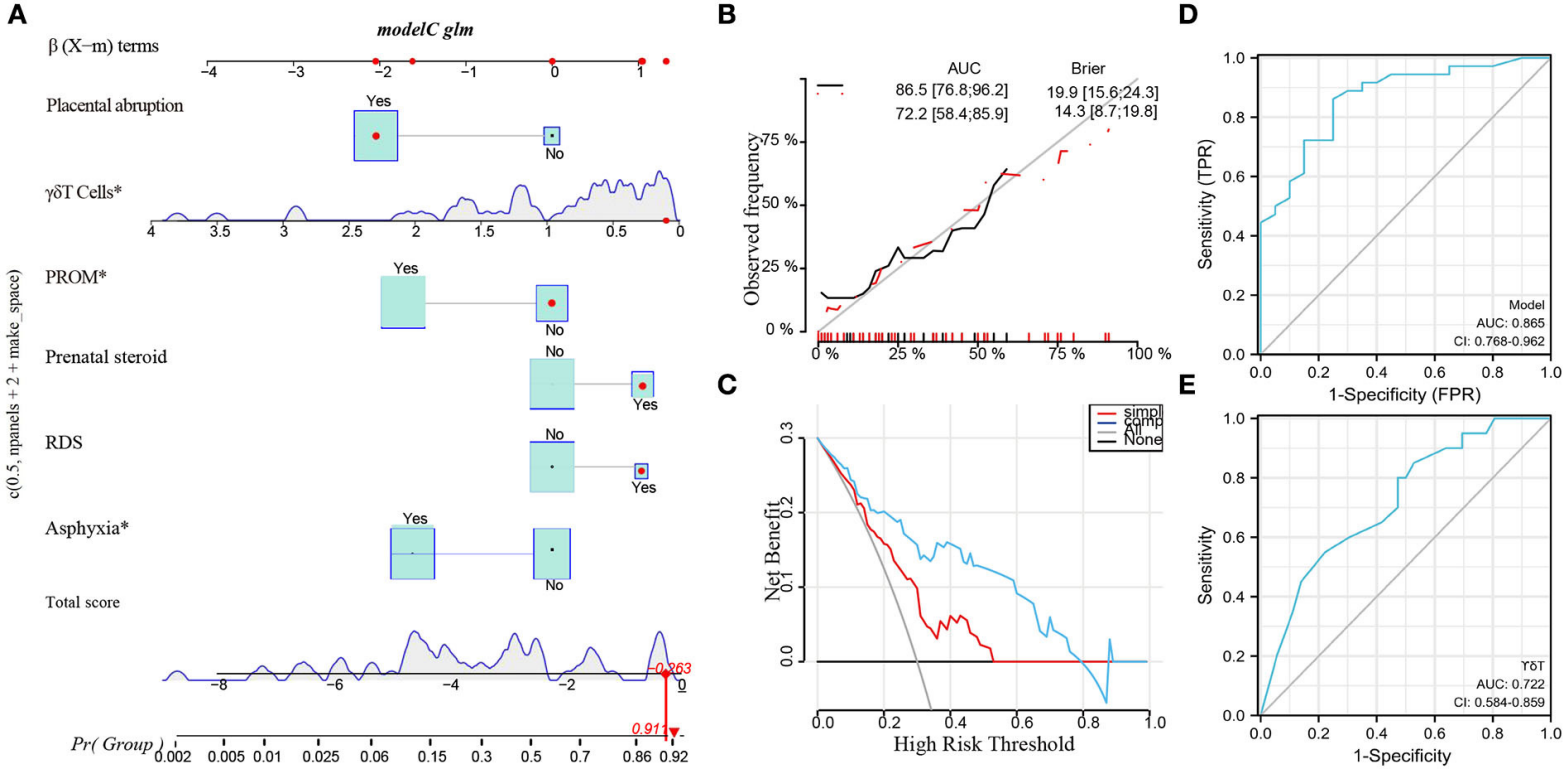
Potential clinical utility of *γδ*T cells in Clinical Applications by Machine Learning. **(A)** Nomogram of the multivariate regression model. **(B)** The calibration curve of a multivariate regression model. **(C)** DCA curve of a multivariate regression model. **(D)** The AUC of ROC curves for the multivariate regression model was 0.865 (***P < 0.000). **(E)** The AUC of ROC analysis of *γδ*T in cPVL was 0.722 (**P < 0.001). PROM, premature rupture of membrane; RDS, respiratory distress syndrome. *P < 0.05.

Based on the above analysis, a nomogram was built (Figure 5A), including the main factors of asphyxia, respiratory distress syndrome, prenatal steroid hormones, premature rupture of membranes, placental abruption, and *γδ*T. The nomogram calibration curves of the probability of cPVL occurrence showed good consistency between prediction and observation in major cohort studies (Figure 5B). The AUC= 0.865, 95% CI (0.768, 0.962), Brier Score = 0.143, 95% CI (8.7, 19.8) were calculated by using the bootstrap method with repeated sampling 1,000 times. The area under the curve (AUC) was calculated, which proved that the certificate nomogram identification and calibration method were reliable. ROC analysis and decision curve analysis (DCA) of nomogram data were performed (Figures 5C,D). The ROC results show that Model 1 AUC = 0.722 and Model 2 AUC = 0.865. The decision curve showed that when the threshold probability of cPVL occurring in the hospital was 0.0 to 0.8, the net benefit level of the application of the nomogram was significantly higher than that of the “no intervention” and “comprehensive intervention” protocols, indicating that the nomogram had good clinical applicability.

### Neurodevelopmental outcomes of study subjects at age 3 years

**Figure 6:**
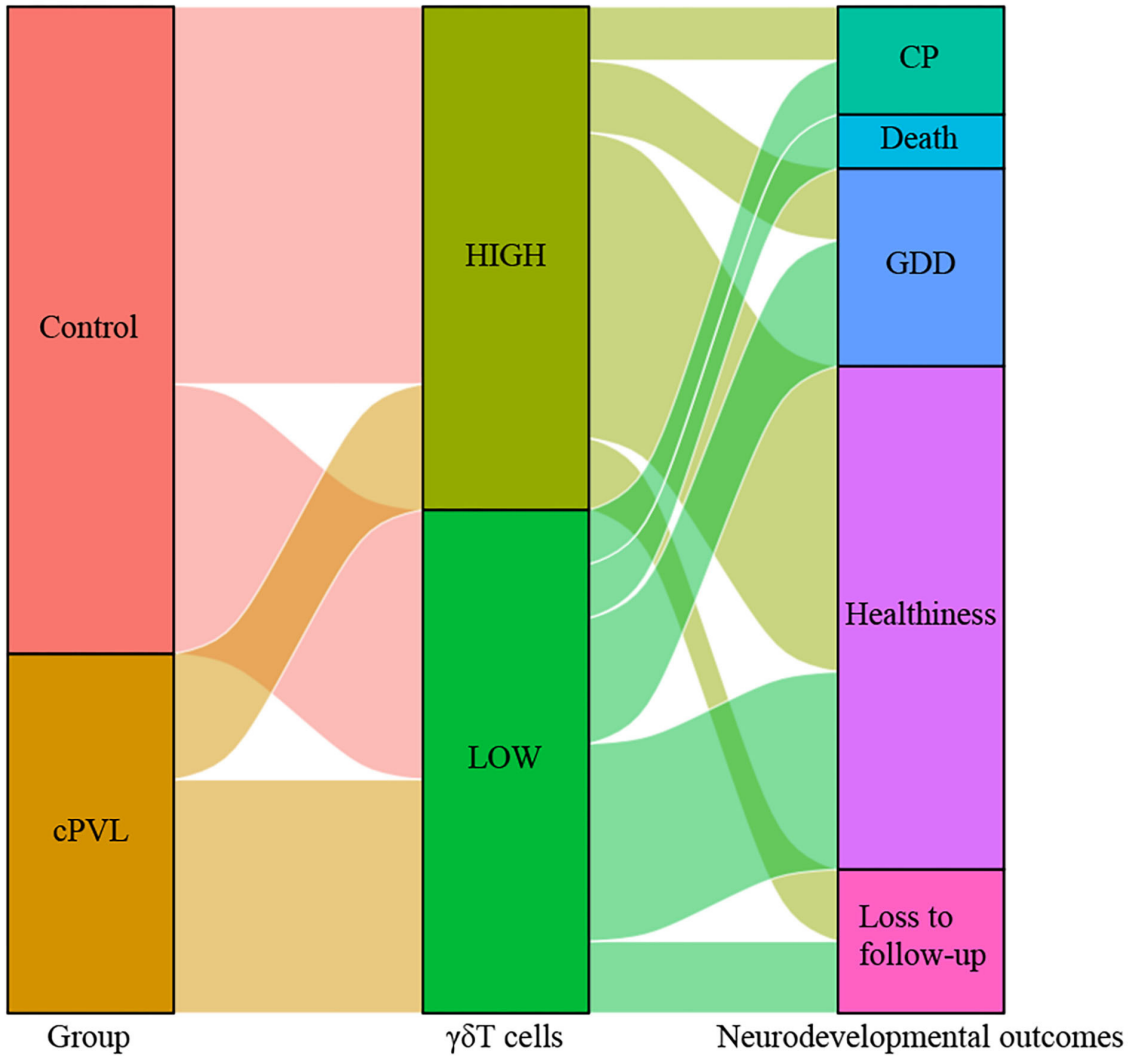
Sankey diagram representing the relationship between *γδ*T cell levels and neurodevelopmental outcomes between the cPVL group and control group. Using the median of *γδ*T cells as a criterion to divide into high and low values to connect two groups of preterm infants and neurodevelopmental outcomes, *γδ*T cell levels can be seen in the Sankey diagram to represent a worse neurodevelopmental outcome. CP, cerebral palsy; GDD, global developmental delay.

Tracking the neurodevelopmental outcomes of these participants at age 3 years was revealed. According to the results of the Gesell scale DQ scores, 56 patients were an average age of 35 ± 5.5 months, 28 cases with normal growth and development (50.0%), 11 cases of growth retardation (19.6%), 6 cases of cerebral palsy (10.7%), 3 cases of death in infancy (5.4%), and 8 cases were lost to follow-up (14.3%). The data were graphed using the Sankey diagram (Figure 6), with the median of *γδ*T cells as a criterion to divide into high and low values, and lower *γδ*T cell levels can be seen in the Sankey diagram to represent a worse neurodevelopmental outcome.

## Discussion

With the improvement of perinatal medicine, the case fatality rate of brain injury in preterm infants has significantly improved, but survivors often develop a variety of severe neurological sequelae (23). Periventricular leukomalacia is one of the leading causes of neurodevelopmental dysfunction in preterm infants, the most severe type of which is cystic peripheral leukomalacia, of which cerebral palsy is the most common sequelae (24, 25). At present, the pathogenesis of brain injury in preterm infants is not fully understood, except that it is related to premature infants' immature development, hypoxia, ischemia, and inflammation may lead to brain injury in preterm infants (8, 26). The cause of cystic periventricular leukomalacia may be related to placental abnormalities and perinatal asphyxia, which is consistent with our findings (27). The diagnosis of brain injury in preterm infants relies primarily on imaging, and due to the lack of typical clinical features in the early stages of the disease, the patients often miss the best opportunity for intervention. The innovative approach to prevent or decrease brain injury in preterm infants requires the discovery of biomarkers able to discriminate infants at risk for injury, monitor the progression of the injury, and assess the efficacy of neuroprotective clinical trials (28). This study investigated the subpopulation of *γδ*T lymphocytes and related cytokine levels in the peripheral blood of preterm infants with brain injury to seek a sensitive and straightforward serum biological marker that reflects brain injury.

*γδ*T cells are the earliest developing cells, and *γδ*T cells precede the development of αβT lymphocytes and are abundant in the first few weeks of fetal development. The potential function of *γδ*T cells is usually pre-programmed during development, rather than spending time on peripheral re-differentiation as traditional αβT cells (29). However, after birth, they make up only a tiny fraction of thymic cells, 0.5–10% of the total number of circulating lymphocytes in adults; they are widely distributed in non-lymphoid tissues and have a variety of activities of innate immune cells (30, 31). It does not have histocompatibility complex (MHC) restriction, which does not require antigen-presenting cells (APCs), and can directly recognize and bind to antigen molecules and lyse target cells (32). Therefore, it plays a bridging role in innate and adaptive immunity and can regulate immune responses by producing interleukin 17A (IL-17A), interferon-γ (IFNγ), and tumor necrosis factor-α (TNF-α), thereby helping to limit the spread of injury and promote homeostasis at the site of injury (33, 34). Previous studies have shown that peripheral immune cells such as CD4+T, CD8+T, and CD3+T are closely associated with ischemic brain injury and can infiltrate the brain through periventricular tissue and perivascular spaces (35). Conventional lymphocytes, especially T cells, can contribute to ischemic brain injury through direct damage to neurons *via* the secretion of granules and cytokines, as well as through the activation of microglia, neutrophils, and brain endothelial cells (36). However, *γδ*T cells are an important subset of “unconventional” T lymphocytes, attacking target cells directly through their cytotoxic activity (37). For example, *γδ*T has the unique ability to bridge innate and adaptive immunity while responding to abroad range of tumors (38). They are able to infiltrate human tumors and recognize tumor antigens, secrete cytotoxic molecules such as granzyme and perforin, mount rapid cytokine responses without undergoing clonal expansion, and activate adaptive immune responses (39). Albertsson et al. (17) found that *γδ*T lymphocytes increased significantly near the leuko-softening foci in sepsis-mediated cerebral white matter injury in neonatal rat model and autopsies of preterm infants. Based on the above point of view, we speculated that *γδ*T lymphocytes may be recruited from the peripheral into brain tissue. However, no studies have explored the changes and mechanisms of *γδ*T lymphocytes in peripheral blood in children with white matter injury.

In the present study, we have observed that the levels of *γδ*T cells and total lymphocytes in patients with cPVL were lower than those in the control group. Since cPVL occurs in preterm infants at < 34 weeks, such patients were susceptible to perinatal risk factors such as asphyxia and premature rupture of membranes (7, 22, 40). Further analysis found that asphyxia and premature rupture of membranes were risk factors for the development of cPVL. Furthermore, after adjustment for these two confounding factors, *γδ*T cells still decreased in the cPVL group compared with the control group. One study reported a significant decrease in the number of all *γδ*T cells as sepsis progressed, *γδ*T cells showed decreases at days 2, 3, and 4 from the start of sepsis (41). We found that lower *γδ*T cell levels predicted a worse neurodevelopmental prognosis for the children, which confirmed that early detection of *γδ*T lymphocytes is more valuable than other lymphocyte subsets in the diagnosis of brain injury in preterm infants. However, after adjusting for all confounding variables, the correlation between the decrease in the proportion of *γδ*T and cPVL was no longer significant. As there are many causes for the occurrence of cPVL, a large number of confounding factors exist and interact with each other. We do not have enough sample size, based on this conclusion drawn from the current experiments, which requires further investigation.

Similar to conventional lymphocytes, *γδ*T cells can produce a variety of cytokines and are involved in a variety of inflammatory responses and autoimmune diseases (42, 43). A study has shown that *γδ*T lymphocytes are directly involved in the development of brain injury by releasing anti-inflammatory cytokines in the brain tissue of adult or newborn rats with ischemic brain injury (44). In the presence of immunological disturbance in the CNS, T cells can be neuroprotective in the short term but can become pathogenic when inflammation is not resolved. Regulatory T cells can also exacerbate neuronal loss by affecting CNS-resident microglia phenotype in an antigen-independent manner (45). T-cell subset diversity and effector functions, such as regulatory T cells, are now considered to be an important T-cell subset for IL-10 release (46). IL-10 is an anti-inflammatory cytokine produced mainly by monocytes and to a lesser extent by lymphocytes. Being pleiotropic in nature, it modulates both immune regulation and inflammation (47). *In vitro* and *in vivo* models of ischemic stroke have convincingly directly and indirectly shown IL-10-mediated neuroprotection; similarly, preclinical brain injury models have suggested a neuroprotective role for IL-10 (48). Preterm in the cPVL group in this study had elevated levels of IL-10, but this effect appeared to be dependent on the type of brain injury pathology, with preclinical and clinical studies often producing conflicting results. Although protective against infectious diseases, cytokine production by *γδ*T cells is involved in many immune pathologies and autoimmune diseases when dysregulated (49). Although *in vitro* and *in vivo* models of cerebral ischemia showed IL-10-mediated neuroprotective effects, the role of IL-10 in predicting clinical outcomes was unclear (50). In limited clinical studies, higher levels of IL-10 after injury were associated with poorer outcomes (51); however, the role of IL-10 in cPVL and its impact on prognosis need to be further investigated.

There is evidence that both systemic and neuroinflammation are clearly associated with preterm brain damage. IL-8, a chemokine, has been implicated as a marker of adverse neurological outcomes in preterm newborns (52, 53). Leviton et al. explored the relationship between the serum IL-8 level in newborns and the occurrence of intracranial hemorrhage, leukoplakia lesions, and cerebral softening in preterm infants. They found that the increase in IL-8 levels lasting more than 1 day was significantly related to intracranial hemorrhage and subsequent white matter injury in healthy controls (54). In addition, studies have shown that elevated maternal IL-8 during pregnancy is associated with altered brain structure and increased schizophrenia in offspring (55). This growing evidence suggests that IL-8 disorders disrupt healthy central nervous system development through various underlying mechanisms during critical periods of neurodevelopment (56). Elevated levels of IL-8 in cord blood have also been shown to be associated with premature rupture of membranes, which increases the risk of brain injury (57). CCL11, which is also referred to as eotaxin-1, belongs to the CC chemokine family known for its role in chemoattracting eosinophils (58), the pro-inflammatory properties of CCL11 which have been reported for the peripheral immune system under various pathophysiological conditions (59). CCL11 is capable of crossing the blood–brain barrier of normal mice, and it is plausible that eotaxins generated in the periphery may exert physiological and pathological actions in the CNS (60). One study reported CCL11 as an important component of the dysregulated inflammatory nerve microenvironment impairing peripheral nerve remyelination (61). Circulating levels of CCL11 also increase significantly after traumatic brain injury in mice, suggesting that CCL11 may play a negative role in functional outcomes after neurological injury by suppressing neurogenesis (62). In our study, the concentration of the inflammatory chemokines IL-8 and eotaxin (CCL11) was elevated in the cPVL group compared with controls, similar to previous studies (63). However, there is still a lack of literature to elucidate the mechanism of serum IL-8 and eotaxin (CCL11) levels involved in the development of cerebral softening in preterm infants.

Unfortunately, this study showed that for preterm infants with small gestational age, IL-17A and IFNγ were below the detection threshold, similarly in the peripheral blood of normal control subjects, and were also difficult to detect. Exploring IL-17A and IFNγ concentrations produced by *γδ*T cells in cPVL peripheral blood requires more sophisticated detection methods. Serum expression of IL-2 and IL-6 in all preterm infants was not detected in this study, so the comparative analysis was not included. A previous study has reported that cytokines or chemokines measured at different gestational ages are not entirely consistent, and serum levels of IL-2, IL-17, IL-6, TNFα, and G-CSF were mostly reduced in preterm infants born at 33 to 35 weeks (64). However, it also shows that in premature infants, their immune response is not yet mature, and the immune state is precarious (65), which needs to be future explored.

In summary, the proportion of *γδ*T lymphocytes in preterm infants was reduced when cystic periventricular leukomalacia occurred, and the levels of serum cytokines IL-8, IL-10, and eotaxin (CCL11) were changed, suggesting that the cellular immune status of preterm infants was disturbed. Peripheral blood *γδ*T cells are correlated with cPVL, which is not only a risk factor for cPVL disease but also neural developmental outcome in preterm infants. However, the chain of causality between cPVL and various lymphocytes remains unclear, which needs to be further studied. Although given the small sample size, high rate of loss to long-term follow-up, the influence of subjective factors on follow-up results, and the lack of objective outcomes such as imaging data and neonatal neurobehavioral measurement scores (NBNA), further studies are needed to assess this possible correlation.

## Data availability statement

The original contributions presented in the study are included in the article/Supplementary material, further inquiries can be directed to the corresponding author/s.

## Ethics statement

This study was approved by the Ethics Committee of The Third Affiliated Hospital of Zhengzhou University (Ethics number: 2021-027-01). Written informed consent to participate in this study was provided by the participants' legal guardian/next of kin.

## Author contributions

MY wrote and edited the manuscript. XJ and FQ collected the data. FX, XZ, and XW designed the project. XJ, FQ, FX, XZ, and XW revised the manuscript. EY and YS gave guidance in flow cytometry. All authors contributed to the article and approved the submitted version.

## Funding

This study was funded by the National Natural Science Foundation: The function and mechanisms of *γδ*T lymphocytes in premature brain injury (general program: 81771418); Additionally, the key Scientific and Technological Project from the Science and Technology Department of Henan Province: Research on Epidemiology and Prevention of Severe Sequelae of Premature infants (172102310497).

## Conflict of interest

The authors declare that the research was conducted in the absence of any commercial or financial relationships that could be construed as a potential conflict of interest.

## Publisher's note

All claims expressed in this article are solely those of the authors and do not necessarily represent those of their affiliated organizations, or those of the publisher, the editors and the reviewers. Any product that may be evaluated in this article, or claim that may be made by its manufacturer, is not guaranteed or endorsed by the publisher.
