# Supplementary material for: The association of γδT lymphocytes with cystic leukomalacia in premature infants
Source: Front Neurol. 2022 Dec 2;13:1043142. doi: 10.3389/fneur.2022.1043142 (PMC9755680; doi:10.3389/fneur.2022.1043142)
Supplement: Supplementary file 1 [file Data_Sheet_1.docx]

**Supplementary Table1. Comparison of the distribution of peripheral blood T lymphocyte subsets of cPVL and control group neonates.**

| Group | control（n=36) | cPVL（n=20) | t/z | *P value* | 95% CI for EXP(B) |
| --- | --- | --- | --- | --- | --- |
| CD4+T cells | 21.72±8.33 | 17.58±9.98 | 1.259 | 0.214 | -1.861-8.14 |
| CD8+T cells | 7.85(5.100,9.825) | 5,75(3.175,8.350) | -1.582 | 0.114 | -0.5-3.9 |
| γδ-T cells* | 1.0(0.5,1.6) | 0.4(0.2,0.8) | -2.734 | 0.006** | 0.1 - 0.9 |
| CD4+/CD8+ | 2.842(2.236,4.066) | 2.696(2.314,3.382) | -0.351 | 0.726 | -0.61-0.829 |
| CD3+T cells | 30.51±12.05 | 23.53±13.79 | 1.937 | 0.054 | -0.114-14.075 |
| Total lymphocytes | 43.00(35.775,54.450) | 36.05(22.75,42.575) | -2.291 | 0.022* | 2.3- 18.0 |

**Supplementary Table2.** **Comparison of the serum cytokine concentrations of cPVL and control** **group neonates.**

| Group | Non PVL（n=30) | PVL（n=7) | t/x2/Z | P value |
| --- | --- | --- | --- | --- |
| IL-8 (CXCL8) | 7.28(16.065,4.550) | 70.20(33.445，168.750） | -2.688 | 0.007** |
| IL-10 | 0.82(0.368,1.023) | 1.70(1.270,2.660) | -2.726 | 0.006** |
| Eotaxin（CCL11） | 3.435（2.530，6.025） | 6.970（5.305，12.065） | -2.023 | 0.043* |
| MCP-1 (CCL2) | 984.06(35.520,122.737) | 164.94(111.375,194.965) | -1.94 | 0.052 |

***: *P* ≤0.001; **: 0.001 <*P* ≤0.01; *: 0.01 <*P* ≤0.05
